# *Carlina vulgaris* L. as a Source of Phytochemicals with Antioxidant Activity

**DOI:** 10.1155/2017/1891849

**Published:** 2017-10-18

**Authors:** Maciej Strzemski, Magdalena Wójciak-Kosior, Ireneusz Sowa, Daniel Załuski, Wojciech Szwerc, Jan Sawicki, Ryszard Kocjan, Marcin Feldo, Sławomir Dresler

**Affiliations:** ^1^Department of Analytical Chemistry, Medical University of Lublin, Chodźki 4a, 20-093 Lublin, Poland; ^2^Department of Pharmacognosy, Ludwik Rydygier Collegium Medicum, Nicolaus Copernicus University, Marie Curie-Skłodowska 9, 85-094 Bydgoszcz, Poland; ^3^Department of Vascular Surgery, Medical University of Lublin, Staszica 11, 20-081 Lublin, Poland; ^4^Department of Plant Physiology, Institute of Biology and Biochemistry, Maria Curie-Skłodowska University, Akademicka 19, 20-033 Lublin, Poland

## Abstract

The methanol extracts from three populations of *Carlina vulgaris* L. were examined for the chlorogenic acid content, mineral content, total phenolic content (TPC), total flavonoid content (TFC), and antioxidant activity. Two populations originated from natural nonmetallicolous habitats (NN (populations from Nasiłów) and NP (populations from Pińczów)), and one metallicolous population (MB) was collected from Bolesław waste heap localized at the place of former open-cast mining of Ag-Pb and Zn-Pb ores dating back to the 13th century and 18th century, respectively. The level of Zn, Pb, Cd, Fe, Ni, and Mn was significantly higher in the root and leaves of MB plants as a result of soil contaminations compared to those of the NN and NP ones. The highest antioxidant potency has been showed by the plants growing in a nonmetallicolous habitat. The flower head extracts obtained from the nonmetallicolous populations also contained the largest amount of chlorogenic acid, whereas the lowest was determined in the roots (ca. 2–3.5 mg/g and 0.2–0.4 mg/g of air-dry weight, resp.). These studies provide important information on the influence of a habitat on the quality of herbal materials and the content of the biologically active primary and secondary metabolites.

## 1. Introduction

Free radicals that are constantly generated in the human body cause oxidative stress. The ratio of free radicals may be increased by the factors such as drugs, chlorinated compounds, deficiency of natural antioxidants, tobacco, and pollution. Despite naturally occurring antioxidant systems in the human body, free radicals cause lipid, protein, and DNA oxidation. These damages at the molecular level may influence the etiology of diseases, such as cancer, atherosclerosis, diabetes, neurodegenerative disorders, and aging-related diseases. Some evidence indicates that the diet rich in antioxidants may be protective against abovementioned diseases [1–3].

One of the special sources of antioxidants is plant-based natural phenolic compounds. Phenolic compounds, known as nonnutritional ingredients in food, constitute one of the most widely occurring groups of phytochemicals with a wide range of physiological properties. They are components of many herbs, fruits, and vegetables, which are associated with the health benefits after their consumption. A clinical trial and epidemiological studies have established that a dietary intake of fruits is strongly associated with a reduced risk of the civilization diseases. In the human body, they play as antiallergenic, antiatherogenic, anti-inflammatory, antimicrobial, antioxidant, and antithrombotic agents. However, their health benefits also depend on their absorption and metabolism, which in turn are determined by their structure, molecular size and solubility in cell wall structure, location of glycosides in cells, and binding of phenolic compounds within the food matrix [4].

Plants belonging to the *Carlina* L. genus (Asteraceae) which comprises over 30 species found in Europe and Asia are rich in antioxidants. Since hundreds of years, some of them have been used in traditional medicine in many countries, for example, in Italy, in Spain, in Hungary, in the Balcan countries, and in Poland. They are used for medicinal and nutritional purposes. They act as diuretic, diaphoretic, stomachic, or antibiotic agents. Extracts are used externally in the treatment of skin diseases. Regarding the chemical composition of *Carlina* spp., reports are scarce and include mainly pentacyclic triterpenes and essential oil. The Strzemski et al.'s previous studies revealed that the *Carlina* spp. contain a high amount of triterpenes (lupeol, lupeol acetate, *α*-amyrin, *β-*amyrin, *β*-amyrin acetate, oleanolic acid, and ursolic acid) [5–9]. There is no information about phenolic acids and minerals and the antioxidant activity of different *Carlina* spp. growing wildly in Poland. As part of a program to search for bioactive constituents from *Carlina* species, this study was focused on the establishment of phenolic compounds (phenolic acids, TPC, and TFC), mineral content, and antioxidant properties of *Carlina vulgaris* L. Moreover, the differences between the populations from a natural habitat and from a postindustrial area contaminated with heavy metals were investigated.

## 2. Experimental

### 2.1. Plant Material

**Figure 1:**
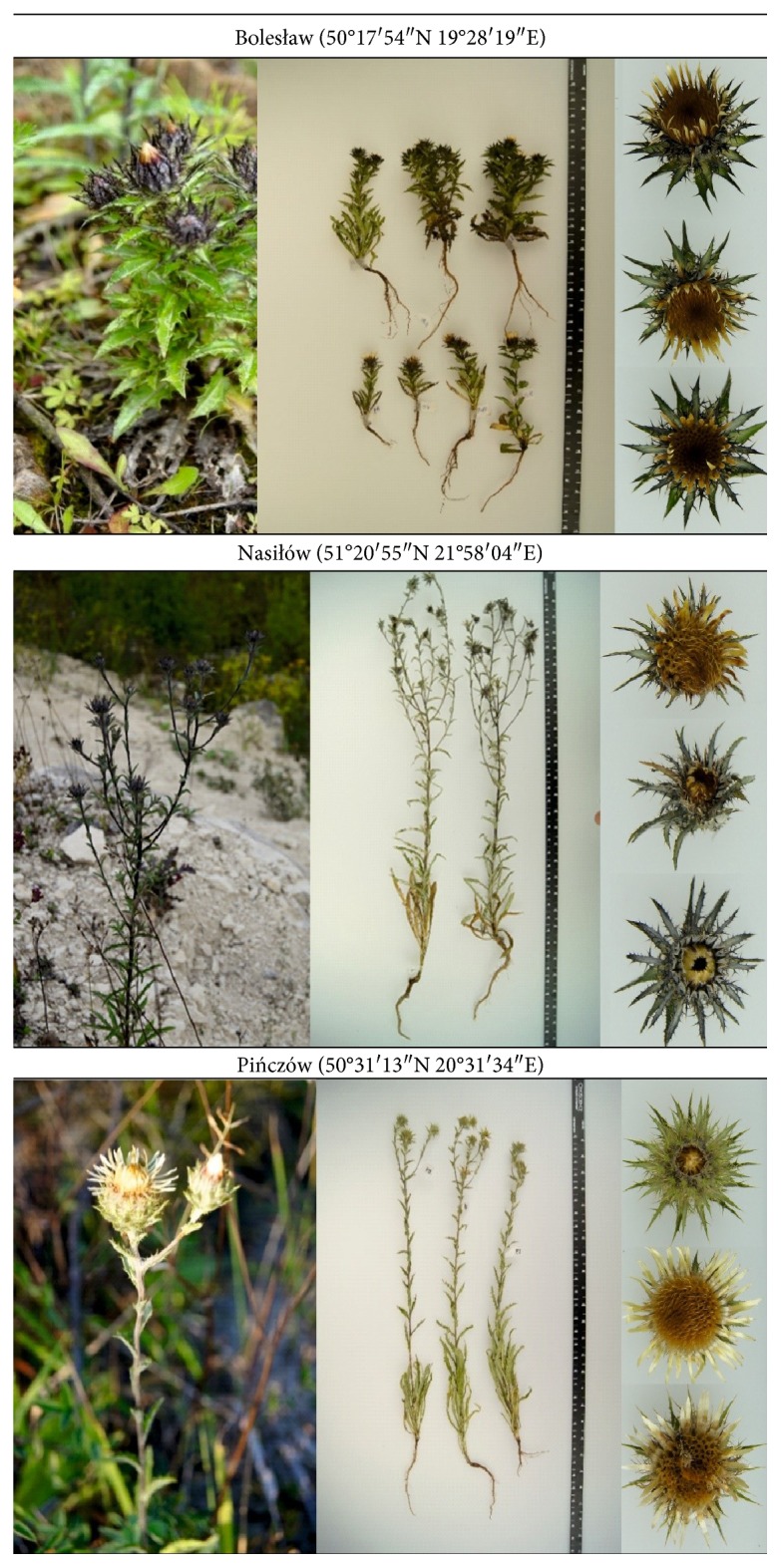
Coordinates of origin sites and exemplary photos of the investigated *Carlina vulgaris* L. plants.

Fifty-two specimens of *Carlina vulgaris* L. (Asteraceae) were collected from three different sites in Poland. Two populations were from Nasiłów (NN, *n* = 22) and Pińczów (NP, *n* = 11), and one population (MB, *n* = 19) originated from a contaminated metalliferous area in Bolesław. The coordinates of sites and exemplary plant photos are presented in Figure 1. The plants were collected at the first half of August 2016. Taxa were identified on the basis of the monographs “*Lebensgeschichte der Gold - und Silberdisteln*” [10] and “*Polish Plants*” [11]. All plant samples were deposited at the Department of Plant Physiology, Institute of Biology and Biochemistry, Maria Curie-Skłodowska University, Poland.

### 2.2. Chemicals and Reagents

Chlorogenic acid standard (≥95%), 2,2′-azino-bis(3-ethylbenzothiazoline-6-sulphonic acid) (ABTS), 2,2-diphenyl-1-picrylhydrazyl (DPPH), Folin–Ciocalteau reagent, trolox, aluminum chloride, suprapure nitric acid (65%), and solution of metal standards (1000 ppm) were purchased from Sigma (St. Louis, MO, USA). Methanol, trifluoroacetic acid (TFA), and HPLC-grade acetonitrile were from Merck (Darmstadt, Germany). Water was deionized and purified by Ultrapure Millipore Direct-Q® 3UV-R (Merck, Darmstadt, Germany).

### 2.3. Analysis of Metal Content

Dried and pulverized roots, leaves, and flower heads (0.1000 g) were mineralized using 10 mL of mixture HNO_3_ : H_2_O (2 : 8, *v*/*v*) in TOPwave apparatus (Analytik Jena AG, Jena, Germany). Mineralization parameters are given in Table S1 in Supplementary Material available online at https://doi.org/10.1155/2017/1891849. The analysis of metal content was conducted using a high-resolution continuum source atomic absorption spectrometer (HR CS AAS) (contrAA® 700, Analytik Jena, Germany) in an electrothermal graphite furnace mode for Ag, Cd, Co, Cr, Cu, Fe, Mn, Mo, Ni, and Pb and using a C_2_H_2_/air flame technique for Zn. The validation parameters are summarized in Table S2.

### 2.4. Extract Preparation

Methanol extracts were prepared according to the previously published procedure [6]. Dried and pulverized roots, leaves, and flower heads (0.5000 g) were extracted four times with methanol (4 × 10 mL) using ultrasonic bath (4 × 15 min). The obtained extracts were combined, concentrated, and filled up with methanol to 10 mL.

### 2.5. Spectroscopic Measurement

The assay was carried out using a Bio-Rad Benchmark Plus microplate spectrometer (Bio-Rad, Hercules, CA, USA). Antioxidant capacities of methanol extracts were determined with the use of 2-azino-bis(3-ethylbenzthiazoline-6-sulphonic acid) (ABTS) and 2,2-diphenyl-1-picrylhydrazyl (DPPH) and expressed as trolox equivalent per gram of air-dry weight (mg TE/g ADW). The total phenolic content (TPC) was established using the Folin–Ciocalteau reagent, and total flavonoid content (TFC) was analyzed based on the reaction with aluminum chloride. TPC and TFC were expressed as equivalent of gallic acid (mg GAE/g ADW) and rutin (mg RUE/g ADW), respectively. All experiments were performed in triplicate.

### 2.6. High-Performance Liquid Chromatography (HPLC)

Chromatographic determination was performed on VWR Hitachi Chromaster 600 chromatograph set coupled with a diode array detector (DAD) (Merck, Darmstadt, Germany) and C18 column Kinetex (10 cm × 4.0 mm i.d., 2.6 *μ*m particle size) (Phenomenex, Torrance, CA, USA). The condition of extract separation was based on literature [12]. A mixture of acetonitrile with 0.025% TFA (solvent A) and water with 0.025% TFA (solvent B) was used as a mobile phase. The gradient program was as follows: 0–8 min (A, 0%; B, 100%), 8–33 min (A, 0–11%; B, 100–89%), 33–38 min (A, 11%; B, 89%), and 38–60 min (A, 11–70%; B, 89–30%). The eluent flow rate was 1.0 mL/min. The column temperature was 25°C. The data were collected in a wavelength range from 200 to 400 nm. The analytes were identified by comparing the retention times and 𝑚/𝑧 values obtained with mass spectrometry (MS) analysis using a micrOTOF-Q II mass spectrometer (Bruker Daltonics, Bremen, Germany). The quantitative analysis was conducted at analytical wavelength characteristic for the investigated compounds using an external calibration method.

### 2.7. Statistical Analysis

Analysis of variance (one-way ANOVA) was applied to the evaluation of difference between the populations. Differences were determined using Fisher's least significance difference test at the 0.05 probability level. Statistical analysis was carried out using Statistica ver. 12 (StatSoft Inc., 2014).

## 3. Results and Discussion

Since the environmental conditions may affect significantly the composition and antioxidant properties of a plant, in our work, the three populations from different sites were investigated. Two of them originated from natural habitats (NN and NP—nonmetallicolous), and one (MB—metallicolous) was collected from Bolesław waste heap localized at the place of former open-cast mining of Ag-Pb and Zn-Pb ores dating back to the13th century and 18th century, respectively. The analysis of soil from Bolesław revealed the increased level of Zn, Cd, and Pb [13], and metal stress may induce [14] or inhibit [15] the production of plant antioxidants. The relationships of variables such as metal content, morphometric parameters, antioxidant activity, and total phenolic and flavonoid content were studied with the use of principal component analysis (PCA).

### 3.1. Metal Content

Microelements and toxic metals were determined in the investigated plant populations. The results are presented in Table 1.

For most metals, the differences of their content between the populations from natural habitats were only slight or statistically insignificant. The amount of Zn, Cr, Mo, Co, and Mn was in the range typically found in plants [16]. However, *C. vulgaris* showed the ability to accumulate Fe and Cu, especially in the root. The level of Fe reached even to 2000 mg/kg and Cu content was above 50 mg/kg, while the values in the other plants usually ranged from 75 to 400 and from 5 to 20 mg/kg, respectively. In turn, the high variation of heavy metal content between the metallicolous population from Bolesław (MB) and the nonmetallicolous populations (NN and NP) has been noticed. As expected, the level of Zn, Pb, Cd, Fe, Ni, and Mn was significantly higher in the root and leaves of MB plants as a result of soil contaminations compared to those of the NN and NP. Particularly, high differences were observed for the content of Pb, Zn, and Cd; the concentration was from several to even few hundredfold higher in the MB population. In MB flower heads, the amount of these metals was also increased; however, it was about 10-fold lower than that in MB leaves and roots. The restricted translocation of heavy metals to generative plant organs is common phenomena occurring in numerous species [16]. The differences in distribution of metals between the MB and NN/NP populations were also observed. In reference populations, the concentration of Cr in roots was 2-3-fold higher than that in leaves whereas accumulation of Zn was higher in leaves. In turn, in MB plants, there were no statistically significant differences between Cr and Zn content in the root and leaves. The translocation of metals in plants exposed to excess of Ni, Pb, and Cd was also reported by Pandey and Sharma [17]. The concentration of Fe was significantly increased in all parts of MB plants compared to those of NP/NN; however, plants are able to accumulate a much higher amount of Fe without a toxic effect [18]. The differences between the amount of Cu and Cr in the metallicolous and NP/NN populations were not statistically significant. For Co and Mo, slight differences were observed; however, it was not linked with postindustrial contamination.

Metal ions have a diverse influence on plants. Cadmium and lead belong to the typical toxic components which cause a cellular damage and disturb cellular homeostasis [17, 19, 20]. Zn, Mn, Fe, Co, and Ni are essential for plant growth and development because they are involved in various physiological processes, such as enzyme activation, absorption, and translocation, and may play an important role in the adaptive responses of plant cells under environmental stresses [16, 21, 22]. They are also essential for human as valuable components of plant-derived products affecting their biological properties.

### 3.2. Morphometric Parameters

**Figure 2:**
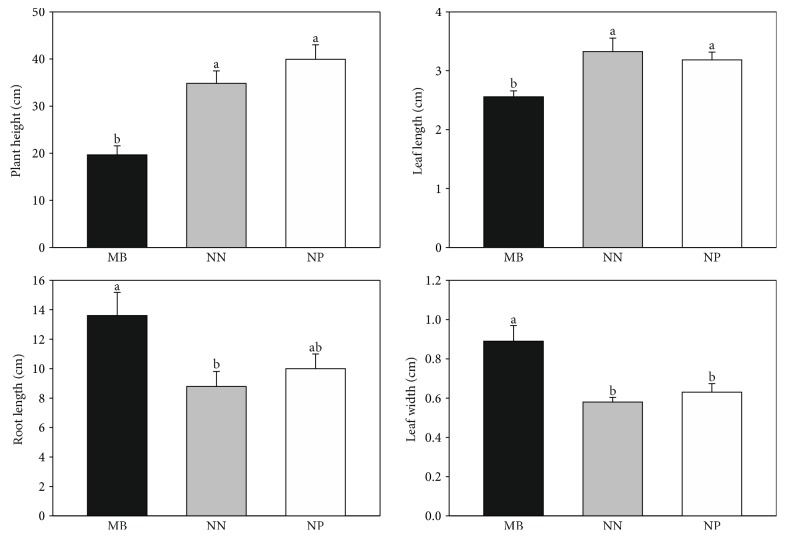
Morphometric parameters obtained for leaves and roots of the investigated *C. vulgaris* populations (NN—from Nasiłów, NP—from Pińczów, and MB—from a metalliferous area in Bolesław). Data are means ± SE. According to Fisher's test (*p* < 0.05), the values followed by different letters are significantly different, the values followed by the same letter are not significantly different, and “ab” indicates that there is no difference between values followed by a and b letters.

The excess of metals in a growth environment may cause the adverse process in a plant, such as chlorosis, necrotic leaf spots, and the other morphological alterations. The tolerance on a decreased level of metals is highly dependent on the plant species, cultivars, or genotypes within a species [23]. In our investigation, the leaf length and width, flower head diameter, number of flower heads and leaves per plant, and root length and plant height were compared between the MB, NN, and NP populations. The results are presented in Figure 2.

The diameter and number of flower heads and leaves were similar for all tested populations (Figure S1); however, the other morphological parameters differed significantly. MB plants were about 2-fold lower, and they had shorter and wilder leaves and longer roots compared to reference populations. The differentiation in morphological features between the metallicolous and NN/NP populations is observed by numerous researchers. For example, in plants exposed to Pb chronic stress, roots are usually longer or/and thicker and Pb is cumulated in their outer part to prevent generative organs [24, 25]. The content of Cd, Pb, Zn, and Ni in the MB population significantly exceeded the values typically found in the other plants, and it affected the plant morphology.

### 3.3. Antioxidant Activity: Total Phenolic Content (TPC) and Total Flavonoid Content (TFC)

**Figure 3:**
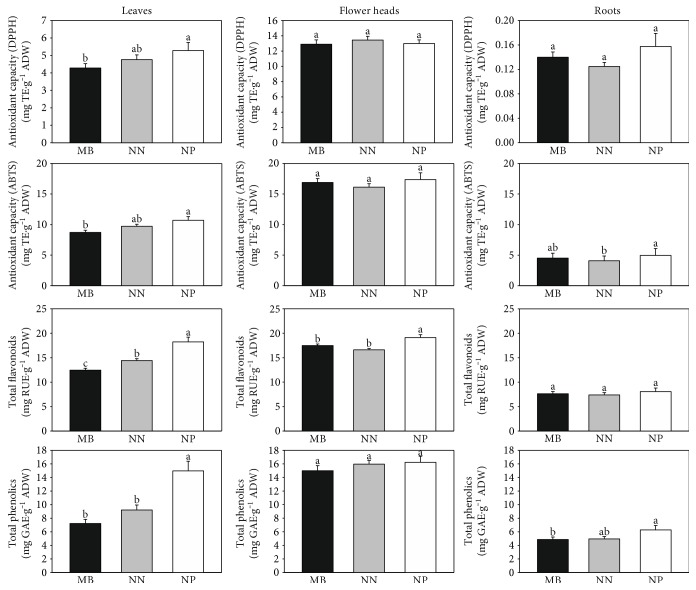
Comparison of antioxidant activity, TPC, and TFC obtained for the different parts of the *C. vulgaris* plant (NN—population from Nasiłów, NP—population from Pińczów, and MB—population from a metalliferous area in Bolesław). Data are means ± SE. According to Fisher's test (*p* < 0.05), the values followed by different letters are significantly different, the values followed by the same letter are not significantly different, and “ab” indicates that there is no difference between values followed by a and b letters.

Plant and plant-based products may be a rich source of polyphenols, which act as antioxidants, and therefore, they are helpful in preventing oxidative stress [1]. The antioxidant activity and the total phenolic and flavonoid content for *C. vulgaris* were established using a spectrophotometric technique. The comparison of results obtained for the different parts of the plant was presented in Figure 3.

As it can be seen, the aboveground part of *C. vulgaris* had significant antioxidant activity. The highest ability to scavenge free radical was noted for flower head extracts, and it was more than 2-fold higher compared to that for the leaf extract. In turn, root extracts exhibited the lowest activity, and it may be explained by generally lower production of antioxidants in the underground part of the plant. No or only slight differences between the investigated populations were observed, and it showed that chronic multimetallic stress had no influence on the antioxidant activity. These findings are in accordance with the results obtained by Dresler et al. [15, 25]. Generally, minor differentiation of TPC and TFC values between the investigated populations was observed for root and flower head extracts. As expected, the highest mean TPC and TFC were obtained for flower heads (15.4 and 18.3 mg/g, resp.) whereas for the root, both values were the lowest (5.8 and 8.2, resp.). The differences between the populations were clearly visible for leaf extracts. The highest TPC and TFC were determined for NP plants; in turn, for the MB population, the values were significantly lower. It suggested that chronic stress decreased the level of polyphenolics. Moreover, the high correlation between TPC/TFC, TPC and DPPH/ABTS, and TFC and DPPH/ABTS was obtained (*r* > 0.79, *r* > 0.61, and *r* > 0.68, resp.). The detailed correlation data are presented in Table S3.

### 3.4. HPLC Analysis

**Figure 4:**
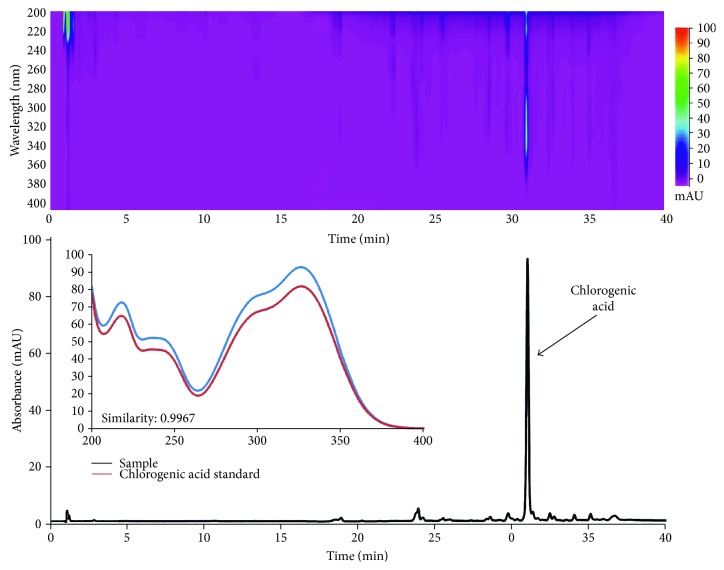
An exemplary 2D and 3D chromatogram of the extract from *C. vulgaris* leaves and UV spectrum of chlorogenic acid standard and compound identified in the extract.

Phenolic acids and their derivatives are considered one of the main groups of plant secondary metabolites with significant antioxidant activity [26]. HPLC of phenolic acids in *C. vulgaris* plant extracts was conducted using an experimentally elaborated gradient elution program which enabled the separation of common plant phenolics [12]. The analysis revealed that chlorogenic acid was a predominant compound found in all parts of the plant. In few samples, caffeic and protocatechuic acids also occurred; however, their contents were slight (below the limit of quantification). The example of the obtained chromatogram is presented in Figure 4.

Quantification of chlorogenic acid was performed based on the linear regression equation (*y* = 124454737.80 × −36813, *r* = 0.9997). The calibration curve was constructed on the basis of the relationship between peak areas and standard concentrations at 5 concentration levels (*n* = 5).

**Figure 5:**
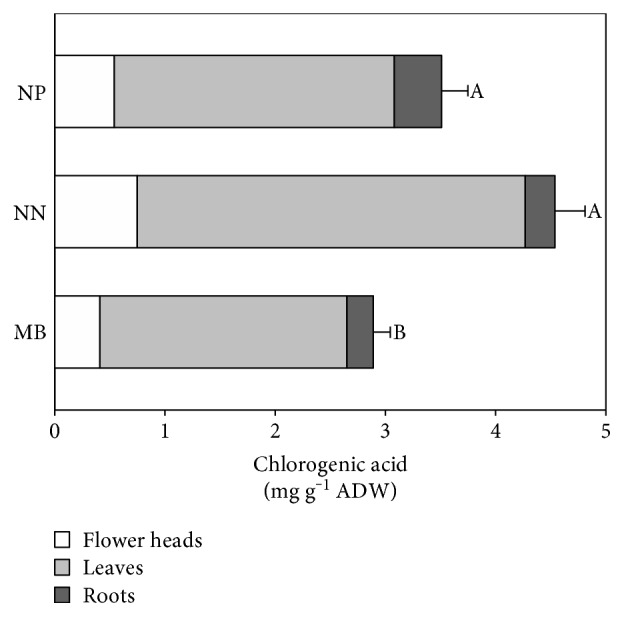
Content of chlorogenic acid in different populations of *C. vulgaris* (NN—population from Nasiłów, NP—population from Pińczów, and MB—population from a metalliferous area in Bolesław). Data are means ± SE. According to Fisher's test (*p* < 0.05), the values followed by different letters are significantly different and the values followed by the same letter are not significantly different.

The results are demonstrated in Figure 5.

Our research revealed that *C. vulgaris* plants are a rich source of chlorogenic acid. Its amount was higher than that in *C. acaulis* and *C. acalifolia* [26]. As it can be seen, the highest content of chlorogenic acid was in the flower heads whereas the lowest was determined in the roots (ca. 2-3.5 mg/g ADW and 0.2–0.4 mg/g ADW, resp.). Moreover, the populations from a natural habitat (NP and NN) contained significantly higher total content of chlorogenic acid compared to the metallicolous population (MB). The differences may be caused by the ability to chelate metal ions by polyphenols with at least two hydroxyl groups in the phenolic ring [27]. Therefore, chlorogenic acid may be bonded in complex, and thus, the amount of its free form is decreased. The antioxidant activity of *C. vulgaris* was statistically significantly correlated with the content of chlorogenic acid; however, the correlation between chlorogenic acid and TPC was lower, and it suggests the presence of other phenolics in plants with lower antioxidant capacity.

### 3.5. Multivariate Comparison between the Investigated Populations

**Figure 6:**
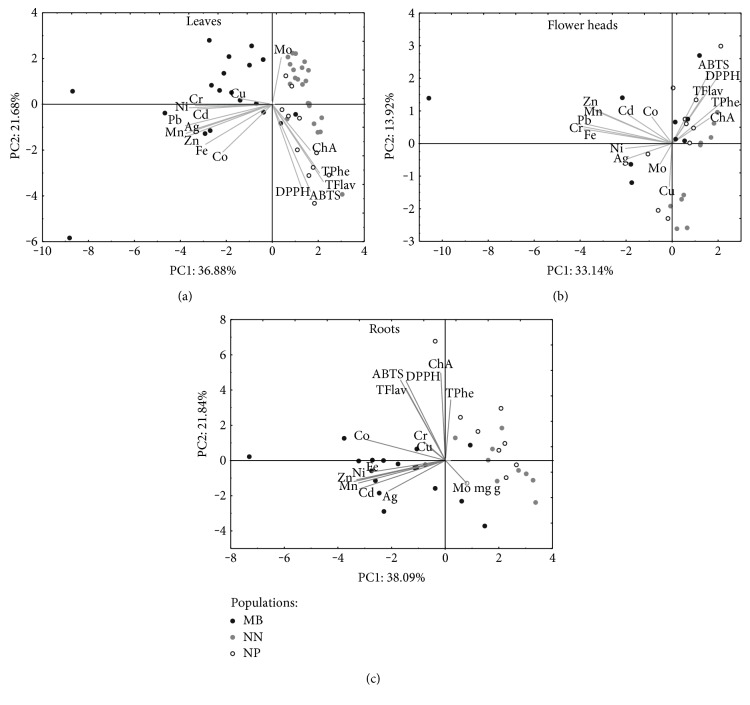
Scatter plot of the PCA of antioxidant capacity, chlorogenic acid concentration (ChA), metal content, and total flavonoid and phenolic content in the investigated populations of *C. vulgaris* (NN—population from Nasiłów, NP—population from Pińczów, and MB—population from a metalliferous area in Bolesław).

The multivariate comparison between the populations is presented in Figure 6.

The performed principal component analysis (PCA) of leaf and root data showed a clear separation between the metallicolous and nonmetallicolous populations (Figures 6(a) and 6(c)). It was noted that the variations among the studied plants were explained by the first two components and represented 60, 46, and 58% of the total variance for leaves, flower heads, and roots, respectively. The first components (in the analysis of all plant organs) were largely negatively determined by heavy metal concentration (except Mo in the leaves and the roots) and these separated plants collected from heavy metal-contaminated and heavy metal-noncontaminated areas (particular leaf and root data analysis) (Figures 5(a) and 5(c)), while PC2 was generally loadings on the secondary metabolites and antioxidant capacity data (positive in the flower heads and roots and negative in the leaf data) and generally showed (leaf data) (Figure 5(a)) difference between the MB and NP populations.

## 4. Conclusion

Our results demonstrate that *C. vulgaris* is rich in polyphenols and minerals. The species growing in noncontaminated areas contain more chlorogenic acid and possess higher antioxidant activity; thus, these species may become ingredients of herbal teas or natural products where a high amount of phytochemicals and minerals is needed. The results obtained in this study confirm an importance of the plants' growth conditions for the safety and quality of the herbal material.

## Supplementary Material

Table S1: Mineralization parameters. Table S2: Validation parameters for AAS analysis. Table S3: Correlation between investigated variables. Figure S1: The diameter and number of flower heads and number of leaves obtained for C. vulgaris populations (NN - from Nasilów; NP - from Pinczów and MB - from metalliferous area in Boleslaw). Data are means ± SE. Values followed by the same letters are not significantly different (p < 0.05).

## Figures and Tables

**Table 1:** Content of metal in leaves, flower heads, and roots of *C. vulgaris* growing in different sites (mg/kg of ADW).

Elements	Leaves			Flower heads			Roots		
	MB	NN	NP	MB	NN	NP	MB	NN	NP
Zn	1458.5 ± 277.7^a^	46.6 ± 4.6^b^	89.0 ± 11.5^b^	100.6 ± 22.8^a^	0.1 ± 0.1^b^	3.3 ± 1.6^b^	1576 ± 249^a^	26.5 ± 6.1^b^	20.5 ± 3.7^b^
Cd	28.74 ± 2.89^a^	0.13 ± 0.09^b^	0.41 ± 0.08^b^	6.59 ± 1.04^a^	0.08 ± 0.01^b^	1.13 ± 1.05^b^	18.73 ± 2.25^a^	0.11 ± 0.02^b^	0.21 ± 0.05^b^
Pb	78.45 ± 21.71^a^	0.73 ± 0.07^b^	6.13 ± 2.56^b^	0.34 ± 0.21^a^	0.07 ± 0.01^a^	0.81 ± 0.51^a^	179.3 ± 17.1^a^	0.97 ± 0.38^b^	1.73 ± 0.41^b^
Cr	1.61 ± 0.44^a^	0.48 ± 0.11^b^	0.74 ± 0.15^ab^	1.40 ± 1.20^a^	0.02 ± 0.02^a^	0.13 ± 0.02^a^	1.78 ± 0.15^a^	2.41 ± 0.43^a^	1.16 ± 0.12^a^
Ni	0.98 ± 0.34^a^	0.03 ± 0.02^b^	0.18 ± 0.06^b^	0.83 ± 0.46^b^	1.11 ± 0.17^ab^	1.67 ± 0.25^a^	1.00 ± 0.09^a^	0.24 ± 0.09^b^	0.09 ± 0.06^b^
Mn	77.54 ± 9.19^a^	10.63 ± 0.61^b^	27.64 ± 3.87^b^	23.30 ± 5.87^a^	4.39 ± 0.42^b^	14.69 ± 2.60^a^	79.59 ± 6.98^a^	19.22 ± 6.88^b^	17.11 ± 3.99b
Mo	1.61 ± 0.37^b^	4.73 ± 0.10^a^	0.05 ± 0.05^b^	0.99 ± 0.34^b^	2.73 ± 0.91^a^	0.23 ± 0.12^b^	36.0 ± 20.4^a^	28.9 ± 12.0^a^	nd^∗^
Co	0.27 ± 0.05^b^	0.12 ± 0.01^c^	0.43 ± 0.08^a^	0.05 ± 0.02^b^	0.03 ± 0.01^b^	0.15 ± 0.04^a^	0.31 ± 0.03^a^	0.13 ± 0.02^b^	0.24 ± 0.04^a^
Cu	45.98 ± 10.22^a^	35.49 ± 3.41^a^	26.18 ± 2.68^a^	43.89 ± 5.10^a^	69.1 ± 21.36^a^	46.82 ± 6.14^a^	67.84 ± 8.79^a^	50.43 ± 5.51^a^	75.9 ± 25.3^a^
Fe	2165.5 ± 516.6^a^	702.3 ± 193.3^b^	353.6 ± 54.0^b^	162.2 ± 79.2^a^	9.5 ± 3.2^b^	8.2 ± 1.4^b^	3945 ± 738^a^	2057 ± 519^b^	989 ± 228^b^
Ag	0.22 ± 0.04^a^	0.06 ± 0.03^b^	0.09 ± 0.03^b^	0.05 ± 0.02^a^	0.07 ± 0.03^a^	0.04 ± 0.01^a^	0.51 ± 0.10^a^	0.03 ± 0.00^b^	0.04 ± 0.02^b^

Means ± SE. According to Fisher's test (*p* < 0.05), the values followed by different letters within the same plant organ and metal are significantly different, the values followed by the same letter are not significantly different, and “ab” indicates that there is no difference between values followed by a and b letters. ^∗^Not detectable.
