# Supplementary material for: Carlina vulgaris L. as a Source of Phytochemicals with Antioxidant Activity
Source: Oxid Med Cell Longev. 2017 Oct 18;2017:1891849. doi: 10.1155/2017/1891849 (PMC5664338; doi:10.1155/2017/1891849)
Supplement: Supplementary file 1 — Table S1: Mineralization parameters. Table S2: Validation parameters for AAS analysis. Table S3: Correlation between investigated variables. Figure S1: The diameter and number of flower heads and number of leaves obtained for C. vulgaris populations (NN - from Nasilów; NP - from Pinczów and MB - from metalliferous area in Boleslaw). Data are means ± SE. Values followed by the same letters are not significantly different (p < 0.05). [file 1891849.f1.pdf]

SUPPLEMENTARY MATERIAL

Carlina vulgaris L. as a source of phytochemicals with an antioxidant activity

Maciej Strzemski,[1] Magdalena Wójciak-Kosior,[1] Ireneusz Sowa,[1] Daniel Załuski,[2] Wojciech Szwerc,[1] Jan Sawicki,[1] Ryszard Kocjan,[1] Marcin Feldo,[3] and Sławomir Dresler[4]

[1] Department of Analytical Chemistry, Medical University of Lublin, Chodźki 4a, Lublin 20-093, Poland

[2] Department of Pharmacognosy, Ludwik Rydygier Collegium Medicum, Nicolaus Copernicus University, Marie Curie-Skłodowska 9, Bydgoszcz 85-094, Poland

[3] Deptartment of Vascular Surgery Medical University of Lublin, Staszica 11, Lublin 20-081, Poland

[4] Department of Plant Physiology, Institute of Biology and Biochemistry, Maria Curie-Skłodowska University, Akademicka 19, Lublin 20-033, Poland

* Correspondence should be addressed to Maciej Strzemski; maciej.strzemski@poczta.onet.pl and Slawomir Dresler; slawomir.dresler@poczta.umcs.lublin.pl

Abstract:

A public interest in herbal products has increased significantly, especially in the plant-based products used in the traditional medicinal systems. The *Carlina* L. genus has been used for medicinal and nutritional purposes in Italy, Spain, Hungary, the Balcan countries and in Poland. The methanol extracts from three populations of *Carlina vulgaris* L. were examined for the content of chlorogenic acid, minerals, total phenolic content (TPC), and total flavonoid content (TFC). The antioxidant activity was determined using DPPH* (2,2-Diphenyl-1-picrylhydrazyl) and ABTS (2,2'-azino-bis(3-ethylbenzothiazoline-6-sulphonic acid). Two populations originated from natural non-metallicolous habitats (NN - populations from Nasiłów and NP - populations from Pińczów) and one metallicolous (MB) were collected from Bolesław waste heap localized at the place of former open-cast mining of Ag-Pb and Zn-Pb ores dating back to the 13th century and 18th century, respectively. The high variation of

heavy metals content between metallicolous population from Bolesław (MB) and non-metallicolous (NN, NP) has been noticed. The level of Zn, Pb, Cd, Fe, Ni and Mn was significantly higher in root and leaves of MB plants as a result of soil contaminations comparing to the NN and NP ones. The highest ability to free radical scavenging was noted for flower head extracts, which correlates with the highest amount of the TPC and TFC for these plant materials (15.4 and 18.3 mg/g, respectively). The highest antioxidant potency has been showed by the plants growing in a non-metallicolous habitat. The flower head extracts obtained from the non-metallicolous populations also contained the largest amount of chlorogenic acid, whereas the lowest was determined in roots (ca. 2 - 3.5 mg/g and 0.2 - 0.4 mg/g of air-dry weight, respectively). Moreover, population from natural habitats (NP, NN) contained significantly higher total content of chlorogenic acid comparing to metallicolous population (MB). These studies provide an important information on an influence of habitat on the quality of herbal materials and a content of the biologically-active primary and secondary metabolites.

Table S1: Mineralization parameters.

Step	Temperature (°C)	Pressure (Bar)	Ramp (°C)	Time (min)	Power (W)
1	170	50	5	10	1305
2	200	50	1	15	1305
3	50	0	1	10	0
4	50	0	1	10	0
5	50	0	1	1	0

Table S2: Validation parameters for AAS analysis.

Element	Equation of the curve	Coefficient of determination	LOD (µg/L)	LOQ (µg/L)	Characteristic concentration (µg/L)
Ag	$y=-0.000812x^2+0.0316x+0.004797$	0.9998	0.0730	0.2435	0.1474
Cd	$y=(0.018+0.08211x)/(1+0.1234x)$	0.9998	0.0555	0.1851	0.0708
Co	$y=0.01718x+0.0004492$	0.9999	0.0737	0.3081	0.2538
Cr	$y=(0.006104+0.01702x)/(1+0.00332x)$	0.9998	0.1641	0.5471	0.2745
Cu	$y=(0.001887 +0.006155x)/(1+0.0124x)$	0.9999	0.3905	1.4750	0.8071
Fe	$y=(0.1285+0.02807x)/(1+0.01464x)$	0.9999	16.420	61.200	0.3448
Mn	$y=-0.001297x^2+0.07116x+0.003779$	0.9998	0.2115	0.7050	0.0784
Mo	$y=(0.008776+0.001187x)/(1-0.00099x)$	0.9997	2.2460	8.6960	3.4062
Ni	$y=-0.000017x^2 +0.007103x +0.01292$	0.9998	3.5100	12.680	0.7175
Pb	$y=-0.000032x^2+0.009548x +0.002201$	0.9999	0.8266	2.7552	0.5485
Zn	$y=0.2669x^2+0.8058x+0.0002907$	0.9998	165.00	5610.0	8.0902

*$Pd/Mg(NO_3)_2$ matrix modifier was used in all analysis (except Ni).

Table S3: Correlation between investigated variables.

	DPPH	ABTS	TFC	TPC	ChA
			**Leaves**		
**DPPH**	-	0.9017	0.6843	0.6112	0.5897
**ABTS**	0.9017	-	0.7907	0.5745	0.5351
**TFC**	0.6843	0.7907	-	0.7890	-
**TPC**	0.6112	0.5745	0.7890	-	-
**ChA**	0.5897	0.5351	-	-	-
			**Flower heads**		
**DPPH**	-	0.7056	-	0.5177	0.7168
**ABTS**	0.7056	-	0.5830	0.4595	0.5508
**TFC**		0.5830	-	-	-
**TPC**	0.5177	0.4595	-	-	0.4481
**ChA**	0.7168	0.3508	-	0.4481	-
			**Roots**		
**DPPH**	-	0.8526	0.6445	-	0.7985
**ABTS**	0.8526	-	0.7226	0.3654	0.7334
**TFC**	0.6445	0.7226	-	0.4464	0.5205
**TPC**	-	0.3654	0.4464	-	0.3701
**ChA**	0.7985	0.7334	0.5205	0.3701	-

*ChA – chlorogenic acid (mg/g ADW)

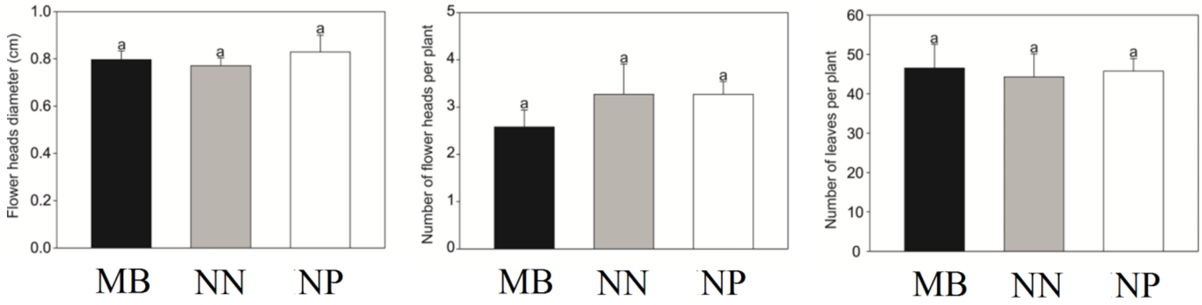

Figure S1: The diameter and number of flower heads and number of leaves obtained for *C. vulgaris* populations (NN - from Nasiłów; NP - from Pińczów and MB - from metalliferous area in Bolesław). Data are means ± SE. Values followed by the same letters are not significantly different ($p < 0.05$).
